# Evaluation of an Oral Subchronic Exposure of Deoxynivalenol on the Composition of Human Gut Microbiota in a Model of Human Microbiota-Associated Rats

**DOI:** 10.1371/journal.pone.0080578

**Published:** 2013-11-18

**Authors:** Manuel J. Saint-Cyr, Agnès Perrin-Guyomard, Paméla Houée, Jean-Guy Rolland, Michel Laurentie

**Affiliations:** 1 Anses, Fougères Laboratory, Antibiotics, Biocides, Residues and Resistance Unit, Fougères, France; 2 Anses, Fougères Laboratory, Scientific Support Unit, Fougères, France; University of California, Los Angeles, United States of America

## Abstract

**Background:**

Deoxynivalenol (DON), a mycotoxin produced by *Fusarium* species, is one of the most prevalent mycotoxins present in cereal crops worldwide. Due to its toxic properties, high stability and prevalence, the presence of DON in the food chain represents a health risk for both humans and animals. The gastrointestinal microbiota represents potentially the first target for these food contaminants. Thus, the effects of mycotoxins on the human gut microbiota is clearly an issue that needs to be addressed in further detail. Using a human microbiota-associated rat model, the aim of the present study was to evaluate the impact of a chronic exposure of DON on the composition of human gut microbiota.

**Methodology/Principal Findings:**

Four groups of 5 germ free male rats each, housed in 4 sterile isolators, were inoculated with a different fresh human fecal flora. Rats were then fed daily by gavage with a solution of DON at 100 µg/kg bw for 4 weeks. Fecal samples were collected at day 0 before the beginning of the treatment; days 7, 16, 21, and 27 during the treatment; and 10 days after the end of the treatment at day 37. DON effect was assessed by real-time PCR quantification of dominant and subdominant bacterial groups in feces. Despite a different intestinal microbiota in each isolator, similar trends were generally observed. During oral DON exposure, a significant increase of 0.5 log10 was observed for the *Bacteroides/Prevotella* group during the first 3 weeks of administration. Concentration levels for *Escherichia coli* decreased at day 27. This significant decrease (0.9 log10 CFU/g) remained stable until the end of the experiment.

**Conclusions/Significance:**

We have demonstrated an impact of oral DON exposure on the human gut microbiota composition. These findings can serve as a template for risk assessment studies of food contaminants on the human gut microbiota.

## Introduction

Deoxynivalenol (DON) is a mycotoxin of the trichothecene family which is a fungal secondary metabolite. Produced by the species *Fusarium*, it is one of the most prevalent mycotoxins present in cereal crops worldwide, and the most frequently occurring type B trichothecene in Europe. A large scale data survey indicated that DON is present in 57% of food samples collected in the European Union [1]. Moreover, the Joint Expert Committee on Food Additives (JECFA) estimates that European cereal consumers have an estimated intake of DON of 1.4 µg/kg body weight (bw) per day [2]. Exposure to DON could also be estimated using biomarkers. Assessment of mycotoxin and its metabolites in urine provide individual data that may establish the prevalence and range of global DON exposure. Therefore, Turner *et al*, have confirmed that French farmers and United Kingdom adults have been exposed to an almost ubiquitous amounts of DON [3]–[5]. Due to its toxic properties, high stability and prevalence, the presence of DON in the food chain represents an important threat to food safety and therefore represents a health risk for both humans and animals [6].

Epidemiological studies linking DON exposure to adverse health outcomes in humans have been reported in China, India, Japan and Korea [7]–[9]. Human gastroenteritis with nausea, diarrhea and vomiting are the main symptoms linked to *Fusarium*-contaminated foods. In addition to the symptoms described in humans, DON toxicity in animals is reflected by feed refusal and consequently growth retardation [10]. At the cellular level, DON has been shown to inhibit protein synthesis and to modulate immune responses [11]. Therefore, a No Observable Adverse Effect Level (NOAEL) has been established at 100 µg/kg of bw based on a decrease body weight gain reported in a 2-year feeding study in mice [12].

Risk assessment approaches to determine a NOAEL are based on physicochemical, pharmacological and toxicological studies. From a microbiological perspective, the ingestion of deoxynivalenol also poses a potential risk to human health since changes in the composition of the human gut microbiota may influence host functions after oral exposure to food contaminants. These changes could, for example, impair colonization resistance that protects the host against pathogen proliferation [13]. Some total diet studies have indicated that dietary exposure through the consumption of food contaminated by mycotoxins is frequent in many populations [14]–[18]. Furthermore, results from Wache *et al*. clearly demonstrated that low doses of DON, which can typically be found in livestock animal feedstuff, had an impact on the swine fecal flora [19]. Despite the fact that the intestine is the major site of DON absorption and the first target of these toxins [20], and the fact that the gastrointestinal tract and its microbiota represent the first barrier against food contaminants, studies describing the effect of DON on the intestinal microbiota are limited. In fact, several *in vitro* studies have identified intestinal and soil borne bacteria that promote metabolism, binding or detoxification of DON [21]–[28]. By contrast, limited data on the impact of DON or other members of the trichothecene family on intestinal microbiota have been published [19], [29]–[32]. Data on the detrimental effects of DON on the human gut microbiota are very limited and therefore, the actual health risk from an oral contamination is unknown. Thus, the effects of mycotoxins on the human gut microbiota are clearly an issue that needs to be addressed in further detail.

Using an *in vivo* approach with a human microbiota-associated rat model (HMA rats), the aim of the present study was to evaluate the impact of a subchronic NOAEL dose exposure of DON on the composition of human gut microbiota. The effect of DON was assessed by monitoring changes in the gut microbiota by real-time PCR (qPCR) quantification of dominant and subdominant bacterial groups.

## Materials and Methods

### Animals

All animal procedures were carried out in strict accordance with the recommendations of the French Ministry of Agriculture. The protocol was approved by Anses's Committee for Ethical Standards and performed in our approved animal breeding facility (Permit Number: D35-137-26).

Axenic Male Sprague-Dawley rats were obtained from the breeding facility of Charles River laboratories (Saint Germain sur l'Arbresle, France). Sterile pelleted feed (SAFE, Scientific Animal Food and Engineering, Augy, France) free of mycotoxin-contamination and sterile water were provided *ad libitum*. The 20 rats (8 weeks old, 120–150 g bw) were housed individually in polycarbonate cages in 4 sterile isolators. Animals were acclimatized for one week.

### Human donors

The procedure of feces sampling in humans does not require unusual and invasive procedures for monitoring and diagnostic, and consequently no ethical permission was mandatory according to the legislation applicable in France in the article of Act L. 1121-1 of 2012. The volunteers signed a consent form for sample utilization and data publication.

The fecal inocula were obtained from 4 healthy adult individuals. These volunteers consumed an unrestricted western-type diet and were not under antibiotic treatment or taking any other drugs known to influence the fecal microbiota composition for at least three months prior to sampling. All subjects were free of known metabolic or gastrointestinal diseases.

### Study design

#### Transfer of human flora into germ-free rats

Four groups of 5 rats were inoculated with a different fresh human fecal flora. The human fecal specimens were collected and immediately placed in an anaerobic atmosphere (GasPak EZ Anaerobe, BD Diagnostic Systems). In the laboratory, samples were transferred to the Whitley A35 Anaerobic Workstation (AES CHEMUNEX, Bruz, France) for microbiological preparation. Fecal samples from the donors were diluted 1/99 (weight/volume) in prereduced Thioglycollate broth with Resazurine and then given orally to the rats in a volume of 1 ml per rat.

#### Treatment with deoxynivalenol

After allowing two weeks for microbiota stabilization, 100 µg/kg bw of DON was administrated daily by gavage to the 4 groups of rats for 4 weeks. Fecal samples were collected at day 0 before the beginning of the treatment; days 7, 16, 21, and 27 during the treatment; and 10 days after the end of the treatment at day 37. After collection, they were stored at −80°C and at −20°C until molecular and physicochemical analyses respectively.

### Chemical reagents

Deoxynivalenol was purchased from Sigma-Aldrich (Saint-Quentin Fallavier, France) and dissolved in acetonitrile (Sigma-Aldrich) at 1 mg/ml. This solution was stored for a maximum of 1 year at −18°C. The working solutions were diluted in physiological saline solution (B. Braun Avitum, Gradignan, France), stored at room temperature and renewed weekly. Their stability was verified, prior to start the animal experiments, by dosing a working DON solution stored in isolator for 2 weeks. DON concentrations were assessed one time, each week, by an in-house developed and validated High Performance Liquid Chromatography with Ultraviolet detection (HPLC–UV) method.

### Bacterial strains and growth

Bacterial type strains used for standard genomic DNA were obtained from the Biological Resource Center of the Institut Pasteur (CRBIP, Paris, France) or Leibniz Institute DSMZ-German Collection of Microorganisms and Cell Cultures (Deutsche Sammlung von Mikroorganismen und Zellkulturen GmbH, Braunschweig, Germany) and are presented in table 1.

**Table 1:** Target organisms, type strains, oligonucleotide primers and probes used in this assay.

Target organism	Type strain	Primer and probe	Sequence 5′ - 3′	References
*All bacteria*	*Escherichia coli* CIP 54.8	F_Bact 1369	CGGTGAATACGTTCCCGG	[33]
		R_Prok 1492	TACGGCTACCTTGTTACGACTT	
		P_TM1389F	6FAM-CTTGTACACACCGCCCGTC	
*Bacteroides/Prevotella* group	*Bacteroides fragilis* CIP 77.16	F_Bacter 11	CCTWCGATGGATAGGGGTT	[33]
		F_Bacter 08	CACGCTACTTGGCTGGTTCAG	
		P_Bac303	VIC-AAGGTCCCCCACATTG	
*Clostridium coccoides* group	*Blautia coccoides* DSM-935	F_Ccoc 07	GACGCCGCGTGAAGGA	[33]
		R_Ccoc 14	AGCCCCAGCCTTTCACATC	
		P_Erec482	VIC-CGGTACCTGACTAAGAAG	
*Clostridium leptum* group	*Clostridium leptum* DSM-753	F_Clept 09	CCTTCCGTGCCGSAGTTA	[33]
		R_Clept 08	GAATTAAACCACATACTCCACTGCTT	
		P_Clep 01	6FAM-CACAATAAGTAATCCACC	
Genus *Bifidobacterium*	*Bifidobacterium adolescentis* CIP 64.59	F_Bifid 09c	CGGGTGAGTAATGCGTGACC	[33]
		R_Bifid 06	TGATAGGACGCGACCCCA	
		P_Bifid	6FAM-CTCCTGGAAACGGGTG	
Genus *Enterococcus*	*Enterococcus faecium* CIP 103014	F_Entero	CCCTTATTGTTAGTTGCCATCATT	[61]
		R_Entero	ACTCGTTGTACTTCCCATTGT	
*Escherichia coli*	*Escherichia coli* CIP 54.8	E.coli F	CATGCCGCGTGTATGAAGAA	[62]
		E.coli R	CGGGTAACGTCAATGAGCAAA	
*Lactobacillus/Leuconostoc/Pediococcus* group	*Lactobacillus acidophilus* DSM-20079	F_Lacto 05	AGCAGTAGGGAATCTTCCA	[33]
		R_Lacto 04	CGCCACTGGTGTTCYTCCATATA	

All strains were inoculated into Tryptic Soy broth, MRS broth and Thioglycollate broth with Resazurine for aerobic, *Lactobacillus acidophilus* and other anaerobic bacteria respectively. Pure cultures were incubated at 37°C in an aerobic or anaerobic atmosphere (10% H2, 10% CO2, 80% N2). The total number of Colony Forming Units (CFU) of each culture was determined by plating 100 µl of the appropriate 10-fold dilution series on Trypticase Soy Agar with 10% Sheep Blood, on MRS Agar and on Schaedler Agar with Vitamin K1 and 5% Sheep Blood (BD Diagnostic Systems, Le Pont de Claix, France) for aerobic, *Lactobacillus acidophilus* and anaerobic bacteria respectively.

### DNA extraction

Genomic DNA from bacterial cultures was extracted using Wizard Genomic DNA Purification Kit (Promega, Charbonnières Les Bains, France) according to the manufacturer's instructions. Extracted DNA was quantified using a BioSpec-nano (Shimadzu Scientific Instruments, Columbia, U.S.A.).

Fecal DNA was extracted from the 200 mg aliquots of feces as described previously [33].

### Oligonucleotide primers and probes

Primers and probes used in this study are presented in table 1. TaqMan qPCR was adapted to quantify the *All bacteria* system, the *Bacteroides/Prevotella*, *Clostridium coccoides*, *Clostridium leptum* groups and the genus *Bifidobacterium*. Real-time qPCR using SYBR Green was performed for *Escherichia coli*, the genus *Enterococcus* and the *Lactobacillus/Leuconostoc/Pediococcus* group. Primer and probe specificities were previously assessed by Furet *et al*. [33]. The TaqMan probes were synthesised by Life Technologies (Saint Aubin, France). The primers were purchased from Sigma Aldrich.

### Real-time PCR conditions

PCR was performed in 25 µl PCR volumes containing 15 µl Power SYBR® Green PCR Master Mix 2X (Life Technologies) or TaqMan Universal PCR 2 (Life Technologies), 0.20 µM of each primer, 0.25 µM of each probe and 10 µl of template DNA at the appropriate dilution (inhibition testing section). Natural Multiplate™ Low-Profile 96-Well Unskirted PCR Plates and a Chromo4 LightCycler were used (Bio-rad, Marnes-La-Coquette, France). The cycling program included a 10 min incubation at 95°C followed by 40 cycles consisting of 95°C for 30 s, 60°C for 1 min. For SYBR-Green® amplifications, to improve amplification specificity, a melting curve analysis of the PCR products was performed by ramping the temperature to 95°C for 10 s and back to 50°C for 15 s followed by incremental increases of 0.5°C/s up to 95°C. The cycle threshold (Ct), *i.e.* the number of PCR cycles necessary to reach the threshold fluorescence level, was manually determined for each run by the user. All the samples were analysed in duplicate.

### PCR Setup Controls

Multiple Non Template Controls (NTC) were included in every assay and amplification of all NTC wells invalidated the entire qPCR run, leading to a repeat run.

### Generation of standard curves

Genomic DNA from the different type strains was used to prepare ten-fold dilution series from 0.1 log_10_ to 8 log_10_ CFU equivalent. Sterile water (15 µl) was used as a negative control. A standard curve for each type strain was generated by plotting the Ct against the logarithm of bacterial quantity (log_10_ CFU equivalent) for each run.

### Inhibition Testing

The TaqMan® Exogenous Internal Positive Control Reagents kit (Life Technologies) was used as an exogenous amplification control to check the appropriate dilution used in real-time PCR [34]. This IPC inhibition assay comprises a control qPCR assay in which the IPC DNA is the only amplifiable target performed in the presence of water. This generates a reference Ct value for the IPC amplicon, characteristic of an uninhibited assay. If the water is substituted with DNA from a sample, a shift of greater than one cycle, to a higher Ct and reduced amplification efficiency indicates the presence of PCR inhibitors in the sample. Each qPCR reaction comprised 1X TaqMan Universal PCR 2, 1X Exo IPC Mix, and 5 µl of diluted sample extract or water. Thermal cycling conditions were 1 cycle of 95°C for 3 min, followed by 40 cycles of 95°C for 30 s, 60°C for 1 min.

### Determination of DON concentrations in feces

Concentrations of DON and its main metabolite deepoxy-deoxynivalenol (DOM-1) in feces from donors and rats were analysed by an adapted method from Sørensen and Elbæk (2005) using a Liquid Chromatography coupled with tandem Mass Spectrometry (LC-MS/MS) (Laboratory LDA 22, Ploufragan, France) [35].

### Data normalization

To overcome the fact that fecal samples may contain more or less water, normalization was done by subtracting the log_10_ CFU/g obtained for the “all bacteria” group from the log_10_ CFU/g for the other bacterial groups [33], [36]. The data are presented as the mean of normalized log_10_ value ± standard deviation of colony-forming unit equivalent per gram of fresh feces (log_10_ CFU/g).

### Statistics

Statistical analysis was performed with SYSTAT V.13 (Systat Software, Chicago, USA). Bacterial levels were compared using the analysis of variance (ANOVA) followed by Dunnett's Multiple Comparison test to compare each sampling time during and after the treatment to the sampling time before the treatment (control). DON concentrations in feces were compared using the analysis of variance (ANOVA). P values <0.05 were considered statistically significant.

## Results

### Evaluation of the qPCR performances

PCR efficiencies were calculated from the slopes of the log-linear portion of the standard curves that were run in each plate. The efficiency range was 82–111% corresponding to good amplification efficiency. The dynamic range covered at least 5 log_10_ concentrations of magnitude, and included the expected interval for the target nucleic acids to be quantified.

We assessed the presence of inhibitors in the DNA samples that could interfere with the following qPCR method. One third of the DNA extracts from fecal samples were included in the inhibition assay and results indicated that no inhibition was present in these samples diluted at 10^−4^ and 10^−5^. In contrast, results from samples diluted at 10^−3^ showed shifts of more than 1 Ct with respect to the reference Ct value, indicative of the presence of PCR inhibitors in these samples. DNA extracts were therefore used at the appropriate dilution of 10^−4^.

The limit of quantification was 6 log_10_ CFU/g, corresponding to the lowest value quantified in the fecal samples with good repeatability (<0.5 Ct).

### Establishment of the human gut microbiota in human microbiota-associated rats

**Figure 1:**
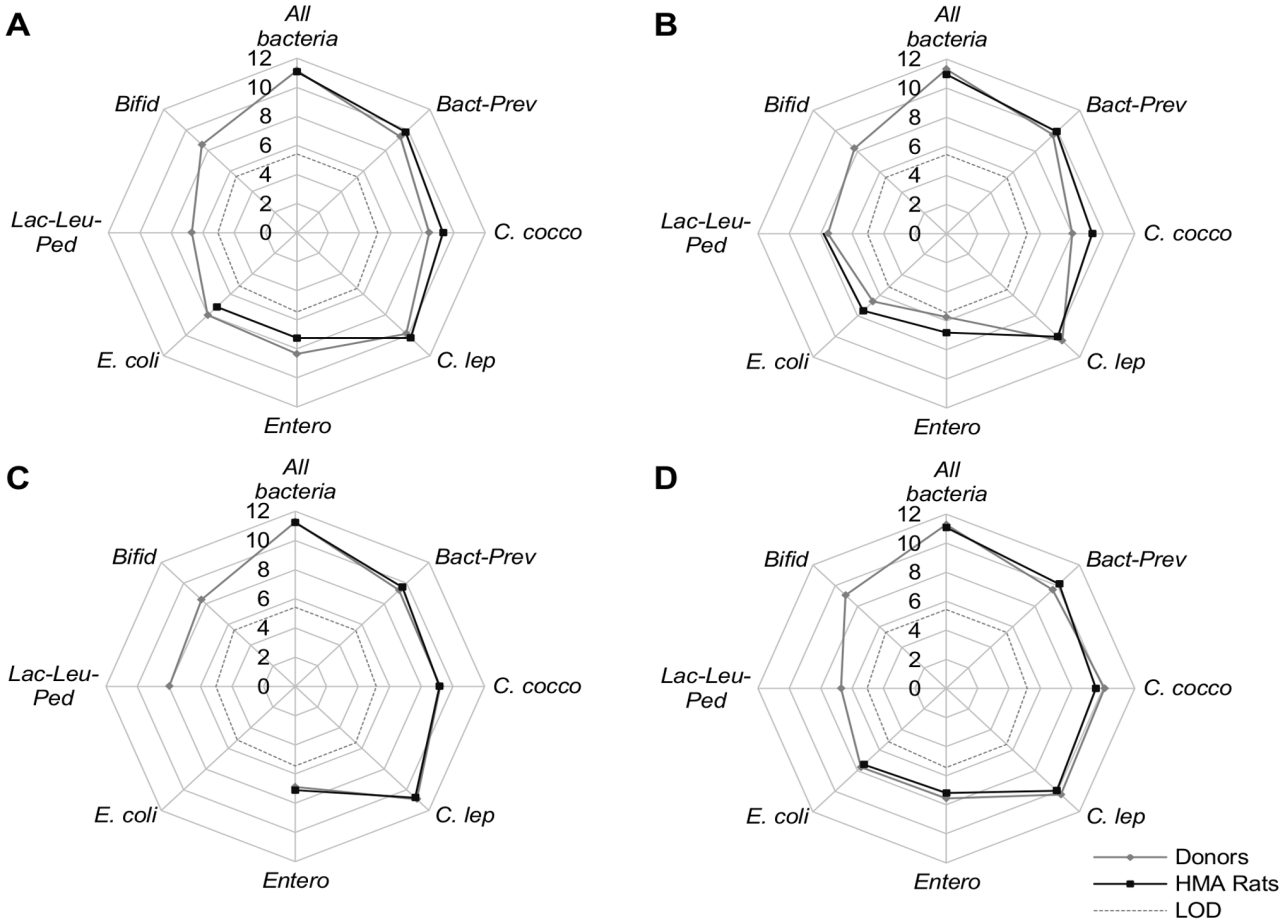
Fecal concentrations of each target bacterial group of the HMA rats and in donors. (A) Isolator I; (B) Isolator II; (C) Isolator III; (D) Isolator IV; Results obtained by qPCR were expressed as the mean of the log_10_ value (for rats n = 5 except for isolator IV where n = 4; for humans n = 2 repetitions) of CFU/g. *Bact-Prev*: *Bacteroides-Prevotella* group; *C. cocco*: *Clostridium coccoides* group; *C. lep*: *Clostridium leptum* group; *Bifid*: Genus *Bifidobacterium*; *Entero*: Genus *Enterococcus*; *E. coli*: *Escherichia coli*; *Lac-Leu-Ped*: *Lactobacillus-Leuconostoc-Pediococcus* group; LOD: limit of detection.

Figure 1 presents the fecal concentration levels for dominant and subdominant bacterial groups in the HMA rats after 2 weeks of stabilization and the corresponding human donors. With the exception of bifidobacteria and the *Lactobacillus/Leuconostoc/Pediococcus* group, all other groups showed concentrations in rat feces close to their concentrations in human feces. These bacteria from human donors established very well in the rat gut tract, in contrast to bifidobacteria and the *Lactobacillus/Leuconostoc/Pediococcus* group which were below the limit of detection of 5.46 and 5.03 log_10_ CFU/g respectively. Our results indicate that these bacterial groups established in rats at lower levels than the inoculating bacterial community from the human donors.

### DON concentrations in feces

DON concentrations in donors' feces were below the limit of detection (3 µg/kg of feces).

In rats, fecal samples from each rat housed in the same isolator were pooled at days 16 and 27. Fecal DON concentrations for each isolator, and for each sampling time, ranged from 240 to 360 µg/kg of feces. Differences in DON concentrations between isolators or sampling time were not significant. Therefore, an overall mean of DON was estimated at 284.4±45.8 µg/kg of feces between day 16 and 27.

DOM-1 was not detected in fecal samples of rats (limit of detection: 3 µg/kg of feces).

### Changes of the gut microbiota in response to DON treatment

Table 2 shows a general overview of the evolution over time of the bacterial groups targeted for each isolator. Despite a different intestinal microbiota in each isolator, similar trends were generally observed for the dominant and subdominant groups targeted in the feces of the humanized rats.

**Table 2:** Time course evolution of bacteria levels in the 4 isolators during the experimental period.

		Treatment (days)					
		Before	During				After
Isolator	Target organism	0	7	16	21	27	37
ISO I	*All bacteria*	11.07±0.14	10.77±0.18	10.82±0.09	10.77±0.26	11.18±0.08	11.10±0.17
	*Bact-Prev*	−1.28±0.15	−0.91±0.11**	−0.79±0.04***	−0.88±0.24**	−1.24±0.12	−1.25±0.19
	*C. cocco*	−1.76±0.30	−1.46±0.33	−1.39±0.44	−2.04±0.69	−2.03±0.36	−1.89±0.78
	*C. lep*	−0.82±0.05	−0.68±0.17	−0.69±0.04	−0.87±0.26	−0.94±0.09	−1.02±0.07
	*Bifid*	< LOD^a^	< LOD^a^	< LOD^a^	< LOD^a^	< LOD^a^	< LOD^a^
	*Entero*	−3.81±0.79	−4.14±0.24	−4.15±0.55	−4.72±0.80	−4.89±0.36	−4.85±0.88
	*E. coli*	−3.84±0.30	−3.60±0.10	−3.74±0.27	−3.97±0.32	−4.94±0.15***	−4.57±0.31***
	*Lac-Leu-Ped*	< LOD^b^	< LOD^b^	< LOD^b^	< LOD^b^	< LOD^b^	< LOD^b^
ISO II	*All bacteria*	10.94±0.10	10.75±0.10	10.74±0.12	10.75±0.07	11.09±0.05	11.10±0.13
	*Bact-Prev*	−1.01±0.18	−0.74±0.11**	−0.69±0.09**	−0.84±0.13	−1.09±0.08	−1.22±0.12*
	*C. cocco*	−1.65±0.15	−1.56±0.25	−1.48±0.32	−1.71±0.42	−1.97±0.25	−2.18±0.13*
	*C. lep*	−0.90±0.21	−0.85±0.11	−0.98±0.06	−1.02±0.09	−0.95±0.11	−0.96±0.14
	*Bifid*	< LOD^a^	< LOD^a^	< LOD^a^	< LOD^a^	< LOD^a^	< LOD^a^
	*Entero*	−4.13±0.88	−4.22±0.60	−4.81±0.50	−4.79±87	−4.83±0.71	−5.01±0.44
	*E. coli*	−3.44±0.48	−3.26±0.16	−3.58±0.16	−3.69±0.40	−4.35±0.41***	−4.38±0.10***
	*Lac-Leu-Ped*	−3.13±1.20	−3.32±1.12	−3.45±1.31	−3.16±1.67	−3.56±1.23	−3.65±1.22
ISO III	*All bacteria*	11.22±0.17	10.68±0.20	10.74±0.15	10.66±0.13	11.30±0.15	11.28±0.11
	*Bact-Prev*	−1.61±0.20	−1.08±0.25***	−1.09±0.11***	−1.08±0.14***	−1.44±0.20	−1.51±0.16
	*C. cocco*	−2.06±0.39	−2.01±0.30	−1.82±0.55	−1.81±0.50	−1.91±0.55	−2.41±0.23
	*C. lep*	−0.45±0.16	−0.21±0.15	−0.15±0.10*	−0.18±0.12*	−0.42±0.20	−0.35±0.11
	*Bifid*	< LOD^a^	< LOD^a^	< LOD^a^	< LOD^a^	< LOD^a^	< LOD^a^
	*Entero*	−4.12±0.48	−4.27±0.62	−4.32±0.72	−4.14±0.77	−4.00±0.38	−4.71±0.27
	*E. coli*	< LOD	< LOD	< LOD	< LOD	< LOD	< LOD
	*Lac-Leu-Ped*	< LOD^b^	< LOD^b^	< LOD^b^	< LOD^b^	< LOD^b^	< LOD^b^
ISO IV	*All bacteria*	11.07±0.14	10.63±0.06	10.63±0.15	10.65±0.11	11.10±0.13	11.20±0.08
	*Bact-Prev*	−0.90±0.09	−0.30±0.16***	−0.30±0.18***	−0.20±0.11***	−0.72±0.16	−0.83±0.14
	*C. cocco*	−1.54±0.23	−0.77±0.47	−1.19±0.63	−1.20±0.64	−1.56±0.44	−1.27±0.36
	*C. lep*	−1.12±0.18	−0.67±0.24	−0.75±0.36	−0.74±0.25	−1.05±0.28	−1.12±0.14
	*Bifid*	< LOD^a^	< LOD^a^	< LOD^a^	< LOD^a^	< LOD^a^	< LOD^a^
	*Entero*	−3.87±0.60	−3.86±0.29	−4.05±0.50	−3.36±0.20	−4.25±0.71	−3.79±0.47
	*E. coli*	−3.66±0.54	−3.31±0.28	−3.59±0.24	−3.46±0.24	−4.03±0.49	−3.93±0.15
	*Lac-Leu-Ped*	< LOD^b^	< LOD^b^	< LOD^b^	< LOD^b^	< LOD^b^	< LOD^b^

Results obtained by qPCR were expressed for all bacteria as the mean of the log_10_ value (n = 5 except for isolator IV where n = 4) ± standard deviation of CFU/g. Normalization was done by subtracting the log_10_ CFU/g obtained for the “all bacteria” group from the log_10_ CFU/g for the other bacterial groups. Results were expressed for the different bacterial groups as the mean of normalized log_10_ value (n = 5 except for isolator IV where n = 4) ± standard deviation of CFU/g.

*P<0.05;

**P<0.01;

***P<0.001.

(a)*Bifid* LOD  =  5.46 log_10_ CFU/g;

(b)Lac-Leu-Ped LOD  =  5.03 log_10_ CFU/g; Bact-Prev: Bacteroides-Prevotella group; C. cocco: Clostridium coccoides group; C. lep: Clostridium leptum group; Bifid: Genus Bifidobacterium; Entero: Genus Enterococcus; E. coli: Escherichia coli; Lac-Leu-Ped: Lactobacillus-Leuconostoc-Pediococcus group.

**Figure 2:**
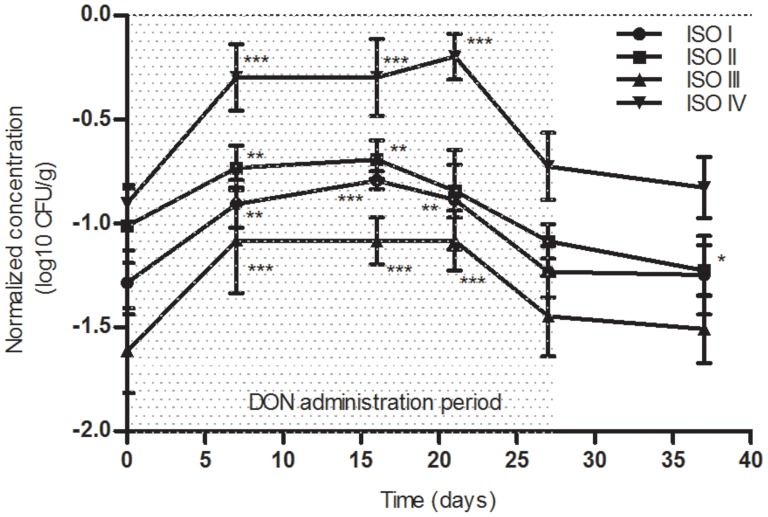
Time course evolution of *Bacteroides-Prevotella* normalized concentrations in the 4 isolators during the experimental period. For the different bacterial groups, results were expressed as the mean of normalized log_10_ value (n = 5 except for isolator IV where n = 4) ± standard deviation of CFU/g.* P<0.05; ** P<0.01; *** P<0.001.

During oral DON exposure, a significant increase of 0.5 log_10_ was observed for the *Bacteroides/Prevotella* group during the first 3 weeks of administration in isolators I, III and IV (P<0.01). Concentrations returned to basal levels similar to that of the control at D0 before the end of the treatment, indicating a transient effect (figure 2).

Concentrations of bifidobacteria and the *Lactobacillus/Leuconostoc/Pediococcus* group remained below the limit of detection of 5.46 and 5.03 log_10_ CFU/g respectively in all DNA extracts. DON seemed to have no effect on these bacterial groups.

During the experimental period, large interindividual fluctuations with SDs higher than 0.5 log_10_ CFU/g were observed in the *Enterococcus* and *Clostridium coccoides* groups.

The *Clostridium leptum* group did not seem to be affected by deoxynivalenol, and remained stable in 3 of the 4 isolators. However, in isolator III, a significant increase of 0.3 log_10_ CFU/g was observed at days 16 and 21 of the treatment (P<0.05).

With the exception of isolator III where *Escherichia coli* was not detected, concentrations of this species decreased at day 27 *i.e.* the end of the DON treatment. This decrease (0.9 log_10_ CFU/g) was significant for isolators I and II (P<0.001). This drop remained stable until the end of the experiment.

## Discussion

In this study, we investigated the effect of an oral subchronic exposure of DON at NOAEL on the intestinal microbiota balance. To the best of our knowledge, this study is the first to analyze the impact of DON on the human intestinal microbiota by real-time PCR in HMA rats.

To avoid ethical issues and long monitoring periods, HMA rodents are widely used to evaluate the effect of contaminants or to elucidate whether the gut commensal microbiota is important for human health [13], [37], [38]. In our study, 5 of 7 dominant (*Bacteroides/Prevotella, Clostridium coccoides*, *Clostridium leptum*, bifidobacteria) and subdominant (*Escherichia coli*, *Lactobacillus/Leuconostoc/Pediococcus Enterococcus*) bacterial groups of human gut microbiota could be successfully established in the germ free rats at levels comparable to the human donors *i.e*. *Bacteroides/Prevotella, Clostridium coccoides* and *Clostridium leptum* groups, *Escherichia coli and* genus *Enterococcus*. However, bifidobacteria and the *Lactobacillus/Leuconostoc/Pediococcus* group were not detected in our experimental conditions. Several studies have previously shown that these bacteria coming from humans decreased in germ free rodents after inoculation, reaching undetectable levels in fecal samples of animals [39]–[42]. Nevertheless, these results demonstrate that the established bacterial community of the feces recipient rats remains similar to the inoculating bacterial community of the human donors. These studies confirm that this animal model is relevant for exploring the effect of food contaminants on the human gut microbiota.

Interactions between bacteria and deoxynivalenol have been demonstrated in several *in vitro* studies dealing with mycotoxin-transforming microorganisms. Several studies have reported transformation of deoxynivalenol by microorganisms from a variety of environmental samples including field soils, wheat leaves and animal gut contents [27], [28], [43]–[46]. This has been assessed with success for potential applications in detoxifying mycotoxins in contaminated food and feed [47]. For example, lactic acid bacteria strains have the ability to remove DON *in vitro* by adsorption of the mycotoxin by the cell wall [48], [49]. This specific binding of DON was able to restrict the consequences of DON on Caco-2 as was shown by Turner *et al*. with the strain *Lactobacillus rhamnosus* [50]. *Eubacterium* sp. is also beneficial in counteracting the toxicity of DON in broilers through the deepoxidization of the mycotoxin in contaminated diets [51]. These observations demonstrate clear interactions between DON and bacteria, which could have impacts on both the bacteria and DON levels. DON absorption is incomplete in the intestine with a bioavailability of about 54% in pigs [52] and 50% in conventional rats (personal data), suggesting that a fraction of ingested DON reaches the colon. This suggestion has been confirmed by Nagl *et al*. in rats, who observed that about 4% of the administered dose of DON were recovered in the feces of animals [53].The quantity of DON that was found in the feces of HMA rats during the treatment suggests that this food contaminant could potentially influence gut microbiota. Indeed, in our study, the significant increase of the *Bacteroides/Prevotella* group and the significant decrease of *Escherichia coli* indicate that DON at the NOAEL (100 µg/kg bw) could induce biological effects on the dynamic of the humanized gut microbiota. Our findings are in accordance with Waché *et al*. who showed changes in the gut microbiota composition of pigs fed with diet naturally contaminated with DON (136 µg/kg bw) [19]. However, these authors reported, using selective media, an increase of aerobic mesophilic bacteria and a decrease of anaerobic sulfite-reducing bacteria during DON treatment [19], which is not consistent with our findings. This difference could be due to the method used to quantify the bacterial population. Indeed, unlike culture-dependent methods, qPCR quantifies all targeted bacteria irrespective of the state (cultivable, viable non-cultivable, non-cultivable and dead bacteria) while conventional microbiological methods only identifies cultivable bacteria (estimated at <30% of the gut microbiota) [54]. In addition, their experimental animal model was the swine where the indigenous gut bacteria are different from those of humans.

In this study, we observed the significant increase of the Bacteroides/Prevotella group. In Human, this shift may be associated to diseases as reviewed by Clemente *et al*. [55]. For example, individuals with Crohn's disease or celiac diseases exhibited a higher abundance of *Bacteroides* than healthy individuals [56], [57].

With the gut microbiota dysbiosis observed in this study, it could be hypothesized that DON may promote the passage of pathogenic micro-organisms present in food and water across the intestinal epithelium. These effects represent a potential health threat as they could contribute to an increase in bacterial infections in animals or humans exposed to DON. This has already been observed by Oswald *et al.* in piglets treated with fumonisin B1 [31], where mycotoxin treatment was associated with an increased bacterial colonization by pathogenic *Escherichia coli* in the intestine of animals.

In addition, DON is known to induce proinflammatory responses in experimental animals [58]. *In vivo* and *in vitro* studies have also shown that immune cells (including macrophages, B and T lymphocytes and natural killer cells) are very sensitive to DON [59], [60]. DON may therefore contribute to modulate infectious diseases through alterations in immune function.

Although this study and others [19], [30] have shown a potential hazard of mycotoxins on the gut microbiota, the NOAEL of these food contaminants is established only on toxicological data. The International Cooperation on Harmonization of Technical Requirements for Veterinary Medicinal Products (VICH) provides a general approach to establishing a microbiological acceptable daily intake (http://www.vichsec.org) to evaluate the safety of residues of veterinary drugs in animal-derived foods for humans. This approach used for veterinary drugs could be extended to mycotoxins which are prevalent food contaminants.

## Conclusions

In conclusion, this study provides data that could help to determine the public health risk of deoxynivalenol on the human gut microbiota.

On one hand, using qPCR we have identified particular genera in human gut microbiota whose concentrations vary after DON exposure at the NOAEL dose. As this effect could have consequences for human health, further investigation would be interesting. Therefore, in order to improve risk assessment in humans, studies on the different functions of the gut microbiota could be performed (barrier effect, enzymatic activities, metabolic profiles) after exposure to DON. Moreover, co-occurrence of mycotoxins is widespread and mycotoxins can be released from their masked forms; therefore, experiments with combinations of mycotoxins and their different forms are necessary and complementary to our study.

On the other hand, we have demonstrated that the NOAEL established for deoxynivalenol based on a toxicological study can have, nonetheless, a microbiologically significant effect by modifying the gut microbiota. Therefore, we suggest that the investigation of the influence of low concentration of mycotoxins on human gut microbiota as a part of the risk assessment process.

Overall, the findings from this investigation could serve as a template for future impact studies of food contaminants on the human gut microbiota.
